# Describing perspectives of health care professionals on active surveillance for the management of prostate cancer

**DOI:** 10.1186/s12913-018-3273-9

**Published:** 2018-06-08

**Authors:** Kittie Pang, Margaret Fitch, Veronique Ouellet, Simone Chevalier, Darrel E. Drachenberg, Antonio Finelli, Jean-Baptiste Lattouf, Alan So, Simon Sutcliffe, Simon Tanguay, Fred Saad, Anne-Marie Mes-Masson

**Affiliations:** 1 Sunnybrook Health Sciences Centre, 2075 Bayview Ave, Toronto, ON Canada; 2 Bloomberg Faculty of Nursing, University of Toronto, Toronto, ON Canada; 3 Institut du cancer de Montréal and Centre de recherche du Centre hospitalier de l’Université de Montréal, 900, St Denis St, Montréal, QC Canada; 4 McGill University and McGill University Health Centre, 1001 Decarie Blvd, Montreal, QC Canada; 5 Manitoba Prostate Centre, 675 McDermot Ave, Winnipeg, MB Canada; 6 University Health Network, 610 University Ave, Toronto, ON Canada; 7 Department of Surgery Université de Montréal, 2900 Edouard Montpetit Blvd, Montreal, QC Canada; 8 Vancouver Prostate Centre, 2660 Oak St, Vancouver, BC Canada; 9 Terry Fox Research Institute, 675 West 10th Avenue, Vancouver, BC Canada; 10 Department of Medicine, Université de Montréal, 2900 Edouard-Montpetit Blvd, Montreal, QC Canada; 11 U of M Endowed Chair in Prostate Cancer, University of Montreal Hospital Center (CHUM), Montreal Cancer Institute/CRCHUM, CHUM - Pavillon R, 900, rue St-Denis, porte R10-464, Montréal, QC H2X 0A9 Canada

**Keywords:** Active surveillance, Prostate cancer, Decision-making, Focus group, Low-risk disease

## Abstract

**Background:**

Over the last decade, active surveillance has proven to be a safe approach for patients with low-risk prostate cancer. Although active surveillance presents several advantages for both patients and the health care system, all eligible patients do not adopt this approach. Our goal was to evaluate the factors that influence physicians to recommend active surveillance and the barriers that impact adherence to this approach.

**Methods:**

Focus groups (*n* = 5) were held with physicians who provided care for men with low-risk prostate cancer and had engaged in conversations with men and their families about active surveillance. The experience of health care professionals (HCPs) was captured to understand their decisions in proposing active surveillance and to reveal the barriers and facilitators that affect the adherence to this approach. A content analysis was performed on the verbatim transcripts from the sessions.

**Results:**

Although physicians agreed that active surveillance is a suitable approach for low-risk prostate cancer patients, they were concerned about the rapidly evolving and non-standardized guidelines for patient follow-up. They pointed out the need for additional tools to appropriately identify proper patients for whom active surveillance is the best option. Urologists and radiation-oncologists were keen to collaborate with each other, but the role of general practitioner remained controversial once patients were referred to a specialist.

**Conclusions:**

Integration of more reliable tools and/or markers in addition to more specific guidelines for patient follow-up would increase the confidence of both patients and physicians in the choice of active surveillance.

**Electronic supplementary material:**

The online version of this article (10.1186/s12913-018-3273-9) contains supplementary material, which is available to authorized users.

## Background

Widespread adoption of prostate-specific antigen (PSA)-based screening for prostate cancer (PCa) has increased the overtreatment of clinically indolent disease, potentially causing more harm than benefit from immediate interventions [1]. Active surveillance (AS) has emerged as a safe primary management strategy to reduce the risk of overtreatment and associated morbidity [2]. Patients eligible for AS undergo continual risk assessment over time until a radical intervention is needed [3]. Despite the feasibility of AS, variability in AS uptake indicates that it is not utilized to its full potential. Moreover, variation in managing patients eligible for AS has been largely attributed to the physician and practice patterns [4]. AS is a multifactorial-based decision that extends beyond disease characteristics and is critically dependent upon the discussion between the patient and the health care professional (HCP) who can significantly influence the final decision [5, 6]. However, even physicians who advocate AS report barriers in convincing patients of the merits of an approach that defers treatment [7].

This study was undertaken to gain a deeper understanding of the perspectives of HCPs regarding AS and the factors they perceive to influence men’s decision to follow this treatment plan. The intention was to describe the emerging practices related to discussion with men about AS. The AS approach is in conflict with the usual message promoted to the public of undergoing curative treatment promptly following a cancer diagnosis, and is often perceived as “doing nothing” during a time of heightened emotional distress for men and their families, which can make new learning and decision-making difficult [8]. Currently, the approaches used by clinicians to inform men and their family members about AS are not defined. Recently, we reported that men required a detailed explanation on AS and its safety as well as guidance in their decision-making [8]. However, there is a lack of standard training to provide HCPs with the support to effectively counsel and advise patients on AS and help with their decision-making. Understanding the perspectives of specialists and general practitioners (GPs), and the approaches they use to discuss with men and their families about AS may highlight barriers that prevent greater uptake of AS and identify areas for improved discussion or communication/education.

## Methods

The study used a qualitative descriptive design to achieve our aim to explore and describe an emerging phenomenon [9]. Focus groups (*n* = 5) were held with HCPs who provided care for men with PCa and engaged in regular conversations with men and their families about AS. Sessions were conducted in four Canadian provinces within academic hospitals: Centre hospitalier de l’Université de Montréal (CHUM), the McGill University Health Centre (MUHC), and the Jewish General Hospital (JGH; participated via videoconference) in Quebec; University Health Network (UHN) in Ontario; Cancer Care in Manitoba; and Vancouver Coastal Health Hospital (VCHH) in British Columbia. Within these centres, PCa care is delivered by inter-professional teams in specialized clinical programs and serve as regional referral centres. To achieve a random sampling, invitation to participate in this study was sent via email to all specialists, fellows and medical students providing care or being trained to care for PCa patients within the studied centres. GPs who referred their patients to the specialists of these centres were also invited via email. To accommodate a physician’s schedule, focus groups were often integrated into regular multi-disciplinary disease/service-specific meetings. The focus group took place between the years 2013 and 2015. Research ethics approval was obtained from each site and participants signed their consent to participate in this study.

A qualitative researcher (M.F.) facilitated all focus group sessions. For the purpose of this study, a focus group guide (Additional file 1: Table S1) was developed through dialogue with the research team. The questions were crafted to explore the following: participants’ views and their understanding regarding the definition of AS, the HCP’s current practice of presenting AS to men and their families, HCPs’ influences on proposing AS to patients, and factors that influence men’s decision-making about adhering and pursuing AS. Most questions were posed as open-ended queries, and probes were inserted only for clarification of any comments made by participants. Sessions were audiotaped and transcribed verbatim for analysis.

**Fig. 1:**
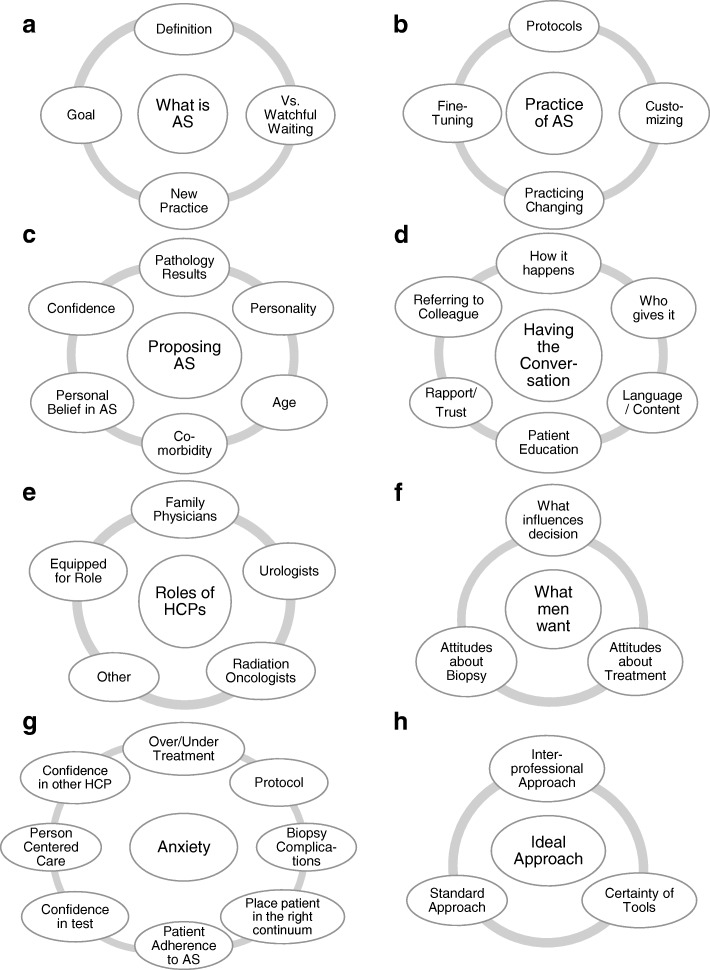
Schematic representation of the content-coding framework used for focus group sessions with health care professionals (HCPs) on active surveillance (AS). Main topics: **a**) What is AS, **b**) Practice of AS, **c**) Proposing AS, **d**) Having the conversation, **e**) Role of HCPs, **f**) What men want, **g**) Anxiety, and **h**) Ideal approach

A conventional content analysis was performed on the verbatim transcripts in order to summarize and describe the various perspectives held by the focus group participants [10]. Four team members (MF, KP, AMM, VO) read transcripts independently, taking marginal notes about the content topics. Together, team members discussed their perspectives about the topics in the transcripts, considered all identified content, and designed a content-coding framework (i.e., topic list and definitions) based on the shared perspectives to achieve consensus. This coding framework is illustrated in Fig. 1. Two members (MF, KP) used the coding framework to code all transcripts from the focus groups and individual interviews using the NVivo software (V10.0 QSR International). The material coded within each of the categories was reviewed in-depth, and the content was summarized for each category with key messages or ideas identified from the participants. The analysis was then presented to three other team members who assessed the clarity and relevance of the findings (two team members had attended group sessions while the other was a clinician highly involved in interactions with men considering AS). This group discussed the analysis and identified overarching ideas across all categories. The resulting consensus on the overarching ideas is the basis for this report.

## Results

The results consist of the key ideas or perspectives shared by the HCPs for each of the following content areas: defining AS, current practices presenting AS to patients and their families, influences on proposing AS to patients, and thoughts about factors that influence men’s decision-making about adhering and pursuing AS. Illustrative quotes are listed per content area in Table 1.

**Table 1:** Illustrative quotes

Content area	Illustrative quotes from health care professionals (HCPs)
HCP perspective defining AS	● When I think of Active Surveillance, I think the term describes the meaning quite thoroughly. So it’s a program of following a patient closely…with the intent to intervene when cure remains a possibility. ● [AS is] the approach of choice when there is little disease, low PSA/Gleason scores, no symptoms. ● It’s a very grey disease. The trajectory is so long, it could be ten, fifteen years before outcome change…I would be surprised you’d see any uniformity, in the way that this is discussed. The way we do it, I think everyone is clear of the standard. How it is done? I think it’s going to be very, very variable.
HCP perspective on AS practice	● The problem is there’s no standard protocol. ● Most of us most probably are not using the same protocol because we adjust for age, like [name] was saying. We sometimes adjust for other pathology; its core for 30 or 40% so I will be more aggressive in doing the biopsy sooner, than the guy who has less than 5%, one of one core, Gleason 6. ● We really don’t have a very good biomarkers or even MRI that have proven to me effective of even efficacious in finding progression. ● Even PSA, you know, multiple groups, Hopkins, UCSF, have shown it is a horrible marker for progression. So the only thing we have right now is, um, biopsy. ● It’s difficult to understand with the protocol, when, um, there’s really no standardize policies that exists outside, you know. Each one publishes their own, but there’s no general agreement of what an ideal protocol is. You are left each one in our guide to our own biases and their own uncertainty and… fear of the disease, you know how close to follow the patient or not. ● And even the protocol at [hospital name] is changing…Even the ones that are doing the most Active Surveillance are constantly changing. ● I think it’s changing year by year as well. So what we are doing now, it’s probably going to be different to what we used to do 5 years ago ● The first couple of years are the easiest part about surveillance. It’s when your practice matures and you are in 5, 10 years and they are sick of the biopsies and they are aware that the PSAs are not a good marker... ● There’s a great deal of learning that we need to, to tap in here and understand about this disease. The transition from Active Surveillance to Watchful Waiting; the challenges of biopsy avoidance to biopsy morbidity…the big issues.
Influences on proposing AS to patients and having the conversation	Factors ● PSA of...depending on the risk of patient... low to intermediate risk. PSA of 10 or less…Gleason of 3 plus 4 or less. ● I think we look at the overall life expectancy, with the age, comorbidities… ● Very much depend on the patient comfort, their psyche, their education, their willingness to actually engage in that dialogue, in their care and um, a cookie cutter doesn’t fit all for sure. ● You know, it’s so variable. You have to individualize, you have to get a sense of the person, the people across the desk from you. You ask them. They have to know what the options are. Having the conversation ● They will have to make it, really informed decision, and they are intelligent and they are educated. I had a guy like that and it took an hour and a half of my time. ● You are there to review their history with them and their journey to date. It possibly reassures them. Changes in medical health, if they are telling me they are unwell, then I start backing off. ● Discussion is very important as is letting the man make his own decision/choice ● The first thing I tell them it’s not a death penalty we are giving them...with this diagnosis. This is a disease that progresses over years and years. Treating it now, you might get cured, but you also get morbidities. And it is tradeoff between treating it now and treating it later. ● It’s rare on the, um, first discussion to be actually conclusive. And I would give them some materials to go home and read and they come back and finalize. ● Patients understand…that we would change according to the follow up. Because if we wouldn’t change our attitude during the follow up, then why would we follow up? ● And I also tell them that sometimes we, we decide to operate or give radiotherapy not because it is very scientific, because it’s not, but because patients change their minds. Patients vary in capacity and desire for information ● Sometimes, these end up to be very complicated and convoluted conversations. Sometimes, they are very easy, it’s very straight forward, you just tell them, nope, no need to do anything, you know, come back in 6 months. Where they go, OK! Fine. See you. So it’s really, very, very much individualized. ● Most of the patients want to understand many of the things, but some just don’t. Some just don’t have capacity and that’s fine too. It’s part of the understanding who you are talking to and you know, how much information do they actually want? Because information overload is very bad as well. So you have to give it in a, presentable manner, with presentable quality that they will respond positively and to understand. So it all depends. Challenges in conversations ● I find it a most difficult conversation, is for the patient, is a marginal candidate for Active Surveillance…so where do you go with that one? ● I mean in these patients, obviously somewhere in between and they are not great candidates for Active Surveillance, on the other hand, they are not…where does this belong, right? ● Often times I find myself explaining things in order for the patient to understand in a very simple manner, but how the disease behaves and we monitor is not simple at all. So I think that’s a big limitation. ● I think what is important from my perspective is that I agree with [doctor’s name] when I talk to the patient. Because when he hears there are two doctors that agree, that I tell him that I agree with …whoever sends me the patient…We might disagree on a small, little details, or this and that. But basically I actually agree, we are saying the same thing. And when they hear that, they relax. GP role ● I would like us to kind of expand a little bit and educate family doctors because they are a great support. ● But mainly …we [GP] know…what prostate cancer is…after the shock of receiving the diagnoses, of course, I understand that they don’t…hear you anymore. So, we can always maybe give the special [talk] My patient would be very, very comfortable to see me on that front. ● The family physician may be consulted by the man in the process of making a final decision: this latter discussion may be very personally focused as the family physician likely knows the individual best; hence it is important that the family physician is aware of active surveillance rationale and is able to explain clearly, and with comfort, why it is a viable option Role of other team members ● We pretty much have the conversation at the same time as the urologist, because most of our urologists send them to us [radiologist] upfront. ● I always offer my patients to see the radiotherapist, or to see another urologists if they want a second opinion. ● We have a nurse that can take him on the side, talk to him about his diagnoses and make him understand there are many prostate cancer and not only one type…she has more time tell him the pros and cons are and teach them every single way to treat. ● Practice here at the Prostate Cancer Centre is that the man has the opportunity to talk with all practitioners ● A specialist in sexuality (nurse specialist) to talk to patients and their partners Ideas for improving the conversation ● We don’t have that nurse set up, which I think it’s valuable. Essentially what the patient needs, it’s time. ● Really to be a good communicator, have good rapport with patients, to actually learn how the communication functions, what are the critical components of communication… is an incredibly valuable skill. ● Here’s a group right in our center, and it the very first question they ask. For me, if I was a prostate cancer patient, I would like to know what other people in my community are doing right now. So I think every center should have a group for support…everything that they could discuss with this patient. I think this will be important... It can help promote Active Surveillance. ● Like the idea of identifying a reliable biomarker; would make it easier to talk with the individual man (relevant information for him) about his risk for progressive disease ● We wouldn’t be in this quandary until we have…be it genetic signature, or some sort of new protein biomarkers… ● I think if you…change the philosophy of the medical community to have Active Surveillance as the default option, you know, that could improve…and you should justify why you should treat. Right now it’s the opposite. That would…change a lot how people embrace it and how often they get raised, you know and that kind of stuff.
HCP perspectives about what men think/want	● Because their immediate reflex is, at least at my practice is, I have cancer why aren’t you removing it? ● So most young patients…all want some sort of treatment. And the patients that go on Active Surveillance are patients that are older, and both the patient and the physician are both very comfortable that the cancer will not catch up with him, through the longevity they have. ● So there are times when active surveillance would have been appropriate but the individual man wants treatment; we need to support their decision ● Some men come with their minds already made up; they have a level of anxiety or concern based on any number of factors (worry about side effects - particularly impotence and incontinence); worry about the cancer bringing about their death, a friend or other family member had prostate cancer as well ● The risk and benefit. I think that’s how the patients make their decisions. So, weight their comfort level with the 5% risk, versus the benefit of avoiding complications. ● I find that…the following 6 months, patients accept more of the idea of being treated than before the resistant…They change their mind. ● The anxiety could be too high, knowing that they live with cancer. ● They like the idea of not necessary being operated or treated with radiation. But the idea with a biopsy, and then another and another biopsy...it’s not an idea that the patients like. ● Most patients are asymptomatic, they are just going on with their lives, they are very happy. And, er, they don’t feel anything. So, unless you have a strong argument that they should be treated, they are very happy not to be treated. ‘Cause you are offering a treatment that is morbid, potentially morbid, versus, er, just being, staying as they are with a few appointments and maybe an unpleasant biopsy down the road.

### Selected demographics

The focus groups involved 48 HCP participants from six different academic centres and affiliated clinics and included GPs, urologists and radiation oncologists (Table 2). Each session lasted between 60 to 90 min. The average age of the participants was 44.6 years (range: 22 to 78 years) and 85% were male. These demographic data were self-reported by the HCPs on the demographic questionnaire designed for the study.

**Table 2:** Breakdown of participant’s^a^ current role by province

Current Role	Quebec^b^	Ontario	Manitoba	British Columbia	Total	%
Urologist and Surgeons	10	6	3	4	23	48.9
Radiation Oncologist	4	0	3	1	8	17.1
General Practitioner	3	0	1	0	4	8.5
Urology Fellow	2	2	0	1	5	10.6
Resident	3	3	0	0	6	12.8

^a^One participant did not state his role

^b^Includes both French and English institutions

### HCP perspectives on defining AS

The participants indicated that AS was an appropriate approach for men with low-risk PCa. They understood that an AS program involved monitoring of the disease on a regular basis with the option for curative treatment if required. AS was seen as providing an option to delay interventional treatment with its inherent side effects, thereby improving patient care and quality of life. It was perceived as the preferred approach for those with low-risk disease and was different from ‘watchful waiting’. They agreed that the latter was used for patients with important comorbidities while AS was recognized as an approach that could prevent unnecessary treatment for clinically insignificant disease and possibly impact costs on the health care system. Illustrative quotes are listed in Table 1.

### HCP perspectives about current AS practice

Participants mentioned that the protocols and practice surrounding AS were changing rapidly. This contributed to challenges in following a protocol since agreed upon guidelines for practice have yet to be developed. In addition, participants expressed the view that once AS was chosen by the patient, the decision was not seen as final. Adjustments could be made when needed during the patient’s clinical course, based on various factors or the individual’s situation.

Participants described the practice surrounding AS and its protocol as varied with differences between institutions and often between specialists or GPs within the same institution. It was noted that the short-term monitoring was fairly consistent, but many participants found it difficult to agree on a protocol for long-term follow-up. They cited the lack of reliable biomarkers and tests, thus, leaving the biopsy as the only feasible test while less invasive but more costly MRIs were not available across sites. Indistinct guidelines on the interpretation of test results were also a cause of confusion among participants and patients, and contributed to variation in practice.

One significant change that specialists have observed since the introduction of AS into practice was the increased amount of time they spent with patients. This time was needed to ensure that proper patient education had been completed and that the patient understood his various treatment options. Patients often returned to their GPs for their opinions regarding treatment options. Some GPs reported they were uncomfortable in discussing AS with patients due to lack of extensive understanding on this treatment choice, while others felt equipped with AS knowledge and believed they could help the patient to make an informed decision about their treatment. GP participants welcomed the notion of additional education sessions to enhance their knowledge on AS and believed it would improve their comfort level in speaking with their patients. Most participants agreed that the practice will likely continue to change as new tests and new tools are developed. Illustrative quotes are enumerated in Table 1.

### Factors influencing discussions about AS with patients

Participants indicated that many factors influenced the specialist’s decision to propose AS to patients (Table 1). However, all participants agreed that they relied heavily on clinical-pathological results (i.e., Gleason Score, PSA blood level, etc.) to determine if AS is suitable for an individual patient. Other factors that impacted their decision to offer AS include the patient’s age, comfort level with the treatment decision, ability to cope, and co-morbidity; these were considered to greater or lesser degree based on the descriptions of the participants in this study. Most did not have a standardized assessment for these factors but based their approach on their clinical acumen.

Participants described PCa disease as a continuum: once AS was undertaken, adjustments could be made by the clinician if some other health issues arose, or if the disease progressed, or if the patient simply had a change of mind. They saw a patient’s decision to choose AS at the time of initial diagnosis as one that could be revisited and changed given new developments. The greatest concern expressed by specialists was for candidates whose clinical profile placed them at the higher risk end of eligibility for AS. They expressed a need for more certainty about placing the patient at the correct point in the disease spectrum and knowing exactly when action was needed based on changing clinical scenarios.

Within the participating centres, the approach to inform men entailed several conversations with the patient and his family, involving various HCPs including urologists, radiation-oncologists and nurses, once the primary HCP determined that AS could be appropriate for the patient. Patients were also free to attend support groups for patients and survivors. There was no standard approach across sites; the number of appointments, the people present during the conversation, topics covered, and support materials provided varied from centre to centre as well as among practitioners within one centre. All participants did not expect a decision on treatment within the initial conversation, and instead, encouraged patients to take their time and inquire about the various treatment options. Most comments reflected the view that patients should make the final decision and emphasized that patients could take their time in making that decision due to the slow-growing nature of their disease.

Each specialty (e.g., urology, radiation-oncology, general practice) plays a different role in the patient’s cancer journey. Participants described their conversations with men as very individualistic and dependent on their own interpretation of the amount of information the patient wanted or needed to know. Based on examples provided by the participants, these conversations ranged from complex and lengthy to very simple and brief, illustrating the variation in approach. Patients were given information about treatment approaches through conversations and in printed materials. However, the support material varied widely across sites. The majority of specialists in academic centres collaborated with each other and felt comfortable in referring and leveraging expertise from other practitioners, but observed challenges in the consistency of messages given to patients by all providers. The GP’s role in the patient decision-making process was also debated as some specialists indicated that the GP’s role should end after the initial referral to the specialist, while others believed that they were a great resource and support for the patient on an on-going basis. Both specialists and GPs agreed that additional education would be beneficial for the GP to confidently support the patient’s decision-making. Participants thought there was room for improvement in holding conversations with men about AS. The availability of other HCPs to augment education and support groups to add support for men were seen as beneficial. Additionally, the development of reliable markers was seen as a priority.

### HCP perspectives about what men think/want

Participants indicated that many factors influenced the patient’s choice, adherence to and continuation of AS over time (Table 1). The factors playing an important role in helping patient’s make their decision included age, personality, the tradeoff between treating now and treating later or not at all, potential side effects, and family history. HCPs perceived that many patients preferred to avoid treatment side effects and were content to remain on an AS protocol as long as they experienced few to no symptoms. Participants reported that signs of disease progression, level of anxiety or a change of mind, and undergoing repeated biopsies were the primary influences in patient’s withdrawal from AS over time. Repeated biopsies negatively impacted AS adherence due to the discomfort experienced from the procedure and potential associated complications.

## Discussion

This study was undertaken to understand the perspectives of specialists and GPs on the practice surrounding AS and associated discussions with men and their families. The practice surrounding discussions about AS with men and their families is still developing and has the potential to be challenging. Following a regime of AS in some ways runs counter to the conventional message about interventional cancer treatment, and how best to present AS information has yet to be described. This study offers a first step in understanding what challenges exist in telling patients about AS.

We examined the factors that influenced the HCP’s decision as to who is offered AS and conversation points with patients and their family, all of which are paramount to improving patient care and their overall quality of life. Although specialists in the study agreed that AS is the most appropriate approach for low-risk PCa, most acknowledged that the lack of standard AS protocol was problematic, an observation already made in the literature [11]. Histological upgrading from Gleason score 6 and above is generally an agreed trigger for intervention and PSA testing is included in most programs but contributes more towards further diagnostic evaluation rather than as a predictor of intervention [11]. This appeared consistent with the views of some but not all participants who did not value PSA as a reliable marker. Participants also recognized that protocols were subject to continual adaptation/change over the years and required an individualized approach. These changing approaches can present a challenge in planning care for patients and helping them understand what will happen.

Currently, the physician’s recommendation has the biggest influence on a patient’s decision to select AS [4, 8, 12]. Variation in AS management is frequently attributed to the physician’s perspectives, practice patterns, or abilities to effectively communicate the merits of AS [13]. Despite their influential role, physicians receive little to no training in counseling patients on AS [7]. In our study, the HCP’s assessment of the patient situation and characteristics drove the conversational approach, yet there was little evidence of a formalized assessment for these factors.

Generally, participants did not use formal instruments or tools for assessing patient characteristics, but relied on clinical-pathological results for baseline criteria, taking age, comorbidity, and the patient’s attitude towards treatment and their coping skills into account. These additional factors have more influence on individualizing care and more impact on the patient’s selection for AS over radical treatment [4, 14]. Our participants tended to be more reluctant to offer AS to younger patients, reflecting a prevailing perception that men with a longer life expectancy have more to lose by delaying curative action, despite enduring greater distress and a poorer quality of life after treatment compared to older patients [4, 15]. Participants also noted that the psychological characteristics of patients influenced their receptivity towards AS. This is in line with literature reports concluding that patients who are anxious or depressed have been reported to be more likely to select radical treatment over AS [4, 16]. Most participants reported a variation in considering all these factors. However, none of them applied systematic decision-making approaches (i.e., decision boards) to assist patients in deciding a course of AS despite the fact that these tools gained favorable attention to communicate patient preferences, improve patient’s understanding of their disease, and reduce decisional regret [12, 17].

Once AS was proposed, some participants believed that an inter-professional team conversation between the HCP, patient and their family was important to assist and support the decision-making. These teams could include nurses, nurse practitioners, primary care providers, and peer support groups. Each of these constituencies would likely offer different types of conversations for men and their partners. Consultation with relevant specialists under a multidisciplinary model of care has been shown to increase AS selection and satisfaction among patients and reduce bias toward the physician’s specialty [6, 18, 19].

Allowing patients to take time for decision-making was highlighted among participants as important for gathering information and considering all options, which was also reported to increase the acceptance of AS [20]. Moreover, men with longer intervals between diagnosis and AS enrollment appeared better adjusted, having had the time to understand the process and their disease, and develop better coping strategies [4]. Guidance towards AS should place greater emphasis on preserving overall health, maintaining functionality and maintaining a quality of life that is at risk after immediate treatment [4]. Importantly, serial biopsies in AS protocols are also associated with discomfort and serious complications that can deter patients from adhering to the program, as noted in our interviews.

In our study, most participants agreed that the development of a standardized approach for AS would be beneficial, given the current variations in terms of protocol, level of patient education materials, methods, and medical community philosophy on AS. Suggested elements that would optimize the AS approach included increasing the accuracy of tests during diagnosis and monitoring to improve the identification of low-risk cancers among all diagnosed patients and reducing overtreatment. At the core of successful AS programs is a strong patient-physician relationship in which patient preferences are recognized, and the risks and benefits of all treatment options are explained clearly and understood by the patient. These insights derived from our study will continue to narrow the information gap that impedes greater AS uptake and contribute to the design of decision aids that will help shared decision-making, improve patient’s understanding, and reduce decisional regret.

### Strength and limitations

One of the strengths of our study is that focus group sessions were held in several Canadian provinces where health care systems are under provincial jurisdiction. It provides a portrait of factors influencing AS uptake by men according to the HCP providing care. Although all provinces were not included, results of this study provide a solid base for developing a questionnaire to survey a larger portion of HCPs in Canada. Capturing a wider set of perspectives could draw on those of other HCPs, physicians and surgeons who practice in non-academic centres and rural settings.

## Conclusion

In this Canadian study, AS was seen as the preferred regimen for men with low-risk PCa. Evidence indicates that this approach can improve patient care and their quality of life, and reduce overtreatment. Currently, various AS protocols are in place across Canada, with more consistent utilization during the initial years following diagnosis. Reliable and consistent tests are required to increase the confidence of providing the right care plan for patients, especially those with test results placing them close to the intermediate risk category. In keeping with the notion of person-centered care, men require tailored approaches to their surveillance and clear explanations to make informed decisions about following AS. There is also considerable appetite from both the HCPs and patients to find less invasive technologies to follow disease progression, and blood-based and imaging approaches could further establish AS as the treatment of choice for men with low-risk disease.

## Additional file

Additional file 1: **Table S1.** Focus group guide**.** General topics and probe associated with each topic to discuss over the focus group session. (DOCX 15 kb)

## Abbreviations

AS: Active surveillance
GP: General practitioner
HCP: Health care professional
PCa: Prostate cancer
PSA: Prostate-specific antigen
