# Supplementary material for: Describing perspectives of health care professionals on active surveillance for the management of prostate cancer
Source: BMC Health Serv Res. 2018 Jun 8;18:430. doi: 10.1186/s12913-018-3273-9 (PMC5994022; doi:10.1186/s12913-018-3273-9)
Supplement: Supplementary file 1 — Table S1. Focus group guide. General topics and probe associated with each topic to discuss over the focus group session. (DOCX 15 kb) [file 12913_2018_3273_MOESM1_ESM.docx]

**Additional file 1: Table 1: Focus Group Guide**

| General topic | Probe |
| --- | --- |
| I would like to understand the term “active surveillance” | - What is your understanding of this term? - How do you define this term? |
| What kind of monitoring occurs for patients who are on active surveillance? | - What kinds of tests? - How often do these tests occur? |
| How do you talk with your patients about active surveillance? | - When does this conversation take place? - How do you explain it to your patients? - What information do you share with them? - Do you have any written information you give to patients? |
| What kinds of questions do patients generally ask you about active surveillance? |  |
| What factors do you think influence the patients’ choice to be on active surveillance? | - Functionality? - Work? Employment? - Family, spouse? - Self-esteem - Philosophy of care; priorities |
| What do you think are the factors that influence patients to remain on or come off active surveillance? |  |
| What influences your decision if a patient is suitable for active surveillance? | - Do you support AS? - Do you recommend it? - What additional information might you need to support AS? |
| Any other comments and/or suggestions? |  |
